# Participatory development and pilot testing of the Makasi intervention: a community-based outreach intervention to improve sub-Saharan and Caribbean immigrants’ empowerment in sexual health

**DOI:** 10.1186/s12889-019-7943-2

**Published:** 2019-12-05

**Authors:** Anne Gosselin, Séverine Carillon, Karna Coulibaly, Valéry Ridde, Corinne Taéron, Veroska Kohou, Iris Zouménou, Romain Mbiribindi, Nicolas Derche, Annabel Desgrées du Loû, for the MAKASI Study Group, Flore Gubert, Maria Melchior, Angèle Delbe, Jacques Ebongue, Fabienne El Khoury, Charles Gaywahali, France Lert, Belinda Lutonadio, Eve Plenel, Laura Maspeyrat, Patricia Mbiribindi, Thierry Miatti, Jean-Paul Ngueya, Andrainolo Ravalihasy, Faya Tess, Jean Voza Lusilu, Iris Zoumenou, and the Makasi Group of Peers

**Affiliations:** 1 French Collaborative Institute on Migrations, Paris, France; 2 Department of Social Epidemiology (ERES), Pierre Louis Institute for Epidemiology and Public Health (IPLESP/ INSERM UMR_S 1136), Paris, France; 3 CEPED, Centre for Population and Development (Paris Descartes University, IRD, Inserm), Paris, France; 4 Solthis, Paris, France; 5 IRD, French Institute for Sustainable Development, Paris, France; 6 ARCAT, Paris, France; 7 Afrique Avenir, Paris, France

**Keywords:** Migrants, Sexual health, Empowerment, Intervention, Community-based research, Sub-Saharan Africa, France

## Abstract

**Background:**

Sub-Saharan and Caribbean immigrants are particularly affected by HIV in Europe, and recent evidence shows that a large portion of them acquired HIV after arrival. There is a need for efficient interventions that can reduce immigrants’ exposure to HIV. We describe the pilot phase of a community-based empowerment outreach intervention among sub-Saharan and Caribbean immigrants in the greater Paris area aimed at 1) constructing the intervention, 2) assessing its feasibility, and 3) assessing the feasibility of its evaluation based on a stepped-wedge approach.

**Methods:**

1) To develop the intervention, a literature review was conducted on existing interventions and participatory approaches developed, including the constitution of peer groups. 2) To assess the intervention’s feasibility, a pilot was conducted between April 2018 and December 2018. A daily register was used to collect data on sociodemographic characteristics of all persons who visited the mobile team to assess eligibility and acceptability. 3) To assess the feasibility of performing a stepped-wedge trial to evaluate the intervention, we compared eligibility, enrolment and retention at 3 months in two arms (immediate vs deferred). Chi-squared tests were used to compare reach and retention between the two arms.

**Results:**

*Intervention development.* The Makasi intervention was designed as an outreach intervention that starts with the persons’ capacities and helps them appropriate existing resources and information and obtain knowledge about sexual health*,* based upon motivational interviewing techniques.

*Intervention Feasibility***.** Between April 2018 and December 2018, a total of 485 persons were identified as eligible. Participation in the intervention was proposed to 79% of eligible persons. When proposed, the persons enrolled in the intervention with a response rate of 69%. Some were lost to follow-up, and 188 persons were finally included.

*Evaluation Feasibility.* The proportions of eligible (45 and 42%) individuals and of enrolled individuals (65 and 74%) were similar and not significantly different in the immediate and deferred arms, respectively.

**Conclusions:**

A community-based outreach intervention aimed at improving sub-Saharan and Caribbean immigrants’ empowerment in sexual health is feasible. The pilot phase was key to identifying challenges, designing a relevant intervention and validating the stepped-wedge protocol for evaluation.

## Background

Sub-Saharan immigrants are particularly affected by HIV in Europe, as they represented 15% of new diagnoses in 2017 [1] and 39% of new diagnoses in France in 2016 [2]. Recent evidence shows that a large part of sub-Saharan African immigrants living with HIV in Europe acquired HIV after arrival in Europe [3], with between one-third and one-half of HIV acquisitions among sub-Saharan immigrants in France occurring post-migration [4]. These HIV acquisitions are primarily linked to experiences of social hardships during settlement [5]. During years without stable housing, resident permits or financial resources, immigrants (i.e., individuals born abroad and who were non-French at birth) are more likely to engage in unprotected sex due to a lack of access to health information and prevention tools, and they are also more likely to experience sexual violence when they are homeless or hosted by someone they know [6, 7]. There are scarce data on immigrants from the Caribbean in France; however, the prevalence of HIV among them is approximately 3% and higher than among sub-Saharans [8], data from the UK suggest that the acquisition of HIV post-migration in this population is important [9]. Despite the lack of specific studies in France on post-migration acquisition of HIV among Caribbean immigrants, they could face the same risk in terms of STIs (Sexually Transmitted Infections). Hence, there is a strong need for effective interventions that can reduce sub-Saharan and Caribbean immigrants’ exposure to HIV and STIs and that take structural difficulties into account.

HIV prevention has experienced important transformations in recent years; the diversified prevention paradigm recommends the use of different prevention tools according to a person’s specific situation, including biomedical prevention tools such as pre-exposure prophylaxis (PrEP). However, at present, biomedical prevention tools are mostly unknown by sub-Saharan immigrants in France, as only 25% of these immigrants had heard about them in a 2016 survey [10], and there is still poor enrolment of immigrants in programmes that give free access to PrEP [11, 12].

Hence, sub-Saharan immigrants’ poor living conditions in France not only increase the risk of HIV infections but also shape immigrants’ access to healthcare and prevention, illustrating how social determinants can play a negative role within different levels of health [13]. Prevention interventions thus need to address both social difficulties and prevention issues and should also reach people who are far from the healthcare system. This analysis was shared by researchers and two non-governmental organisations (NGOs) (1 of which was a community-based organisation, CBO) in the greater Paris area and was the starting point for the Makasi intervention.

When they arrive in France, sub-Saharan immigrants experience long years of social hardship [14, 15]. Although government-sponsored social and sanitary resources to support immigrants exists in France, many immigrants are unaware of available social supports, as information on resources is not adequately disseminated. In addition, the organisation of the social and healthcare system is complex and thus access can be challenging; finally, many immigrants may be too afraid to use available resources for fear of being deported and encountering discrimination based on their immigration status, race or ethnicity [16]. Immigrants face additional barriers to access, such as language barriers or administrative barriers [17, 18]. Caribbean immigrants also encounter difficulties in their settlement in France, and they experience high poverty rates [19]. Eventually, in a nationally representative survey, 47% of sub-Saharan immigrants reported discrimination in France [20]. In this context, obtaining the necessary information and resources to improve one’s social situation and health is not easy. For these reasons, an empowerment framework seemed a highly relevant theoretical framework to developing a new intervention. Empowerment in health can be defined as a psycho-social process that promotes the participation and agency of persons, organisations and communities to improve control over their own health. It refers to both an outcome (people are empowered or not) and to the process of becoming more empowered [21]. The empowerment approach has already been used in different contexts in which people lack some control in their living conditions and their health. Empowerment can then be considered a proximal factor of sexual health, as it was shown to positively impact health behaviours, such as increasing condom use or refusing unsafe sex [22, 23].

However, little evidence exists regarding empowerment in health interventions directed towards immigrants, particularly in the European context. The experience of migration itself is linked to challenging and potentially disempowering experiences. Finding housing and employment are difficult for immigrants, who often must simultaneously seek legal status in the country. We concluded that it would be important in an intervention aimed at empowering immigrants to support them in addressing material needs, while also intervening to facilitate HIV risk reduction. In addition to the previously mentioned interventions among non-immigrants in the United States and India [22–25], there exist interventions among Latino immigrants in the United States [26], who have a very different context in terms of country of origin (i.e., cultural background, perception of HIV) and host country (different organisation of the social and healthcare system). Finally, to create an empowerment intervention adapted to our specific context, it was necessary to engage a community-based approach to design an acceptable, feasible and relevant intervention aimed at empowering sub-Saharan and Caribbean immigrants in the greater Paris area in sexual health. This resultant intervention is called Makasi, which means “strong, resistant” in Lingala, a language spoken in Central Africa.

The purpose of this paper is threefold: 1) to describe the participatory development of a community-based empowerment outreach intervention among immigrants, 2) to assess its feasibility, and 3) to assess the feasibility of evaluating it with a stepped-wedge approach.

## Methods

Here we describe how we developed and assessed feasibility of the intervention. The complete design of the intervention (contents, eligibility criteria, outreach strategies), and the results of the feasibility analysis, are presented in the Results section. All analyses were conducted on data collected between January 2017 and December 2018. The preparatory phase (to develop the intervention) was conducted between January 2017 and March 2018, and the pilot phase (to assess the feasibility of the intervention and the feasibility of an evaluation trial) was conducted from April 2018 to December 2018. Immigrants were defined as individuals born in a country in sub-Saharan Africa or in the non-French Caribbean (mostly Haïti) with a non-French nationality at birth, whatever their current legal situation or nationality may be.

### Developing the Makasi intervention

Two methods were used in parallel during the pilot phase to develop the Makasi intervention.

First, a thorough literature review was conducted using a method of review for effective interventions [27]. The key words used to search for reviews in PubMed were “HIV”, “prevention”, “African”, “Caribbean” and “intervention” (2007–2017). Reviews published on empowerment interventions in peer-reviewed journals as well as in the Cochrane Library, NICE and HAS reviews were read to identify potential existing validated interventions.

Second, participatory approaches were developed. Participatory action research recognises the wealth of assets that community members bring to the processes of knowing, creating knowledge and acting on that knowledge to bring about desired change [28]. Participatory action aims to transform the role of the ones usually acting as subjects of the research and involve them as active researchers. It also makes developing and reflecting on actions part of the research process. The Makasi project involved researchers, 1 CBO (Afrique Avenir), 1 non-governmental organisation (NGO) (Arcat) and peer groups.

The participatory approach was key to the iterative process of defining and revising the intervention that lasted between January 2017 and December 2018. It was implemented through four bodies:

- A community advisory committee was created. It was composed of a group of peers, i.e., immigrants from sub-Saharan Africa or the Caribbean living with HIV who had experienced social hardship and who were beneficiaries of one of the NGOs (Arcat). Participation with this group of peers was proposed to all the NGO beneficiaries fulfilling these criteria. It was explicitly said to all individuals interested in participating that their unwillingness to participate would not impact current or future support or services from the NGO. Reimbursement vouchers were provided for participation in a meeting or activity according to the time spent. The group comprised 14 peers (6 women and 8 men) and was divided into two smaller groups (one morning session and one evening session) so that everyone could attend. The community advisory committee was involved in all the phases of the research; in total, there were 11 meetings to help define the contents of the intervention and then to give feedback on its implementation in the field during the pilot phase. Additionally, two to three members were invited to the meetings of the research group.
- Once the pilot phase began, a weekly field debriefing was started with members of both NGOs who ensured the implementation of the intervention in the field and researchers who were responsible for the feasibility study. During the pilot phase, the debriefing allowed all attendees to report on challenges with both the intervention and the data collection.
- The complete mobile team that was implementing the outreach for the intervention was gathered for a meeting every three months to discuss recruitment and outreach challenges. All the persons on the mobile team were from sub-Saharan Africa or Caribbean immigrant communities.
- The Steering Committee that gathered the researchers and the heads of the two partner NGOs, who made the decisions about the project, met every two months.

### Feasibility study

Once the theory and content of the intervention were defined through the participatory process, a pilot phase was conducted between April 2018 and December 2018 to test the feasibility of the intervention. The test was conducted by the mobile team of Afrique Avenir and the social workers specially hired for the project during nine months in four locations where the mobile team was already intervening with an important concentration of African and Caribbean populations: one location in Paris and three in Seine-Saint-Denis (the greater Paris area). The mobile team used flyers and posters and also proposed the Makasi intervention after administering the rapid HIV test they routinely offered. A daily register was used to collect socio-demographic data on all persons who visited the mobile team. In-person questionnaires about social and family situations, sexual health, mental health, HIV knowledge and testing, and empowerment were conducted pre- and post-intervention.

### Feasibility of a stepped-wedge evaluation

The pilot phase was also used to test the strategy, a stepped wedge design, for evaluating the impact of the intervention. A randomised two-arm design was planned with the day of intervention being randomised between immediate (immediate intervention) or deferred (intervention in three months) to ensure a controlled evaluation of the impact of the intervention after three months between a group who received the intervention and a group who did not. The strategy for the qualitative evaluation was also tested (for the evaluation design, please see Additional file 1).

Indicators of feasibility of a stepped wedge design were the reach and retention in each arm of the study. Reach was defined as the proportion of eligible individuals who agreed to participate and were enrolled; as well, we defined retention as the proportion of those enrolled who were retained three months after the baseline enrollment. Chi-squared tests were used to compare reach and retention between the two arms in the study (immediate vs deferred).

### Reflexive process

As noted by Tremblay and Parent, reflexivity is necessary for population health intervention research. According to those authors, reflexivity can be defined as ‘*an intended and conscious intellectual activity in which individuals (or groups) explore or examine their experiences to develop new understandings that ultimately shape their actions”* [29]*.* A series of reflexive workshops (3 workshops between December 2018 and January 2019) was conducted at the end of the pilot phase, in which all the members of the project team were gathered: researchers, operational teams and peers. The purpose of a reflexive workshop is to enable individuals to produce a retrospective and reflexive analysis of an action implementation [30]. The challenges faced by the team after a nine-month test included how to include all the persons who accepted, the inclusion rate being too slow, and low retention at three months, and after a presentation of these challenges, a collective brainstorming session with Post-it notes took place, in which every person proposed three ideas to address the challenges and put them on a board. Then, an organiser would pick up several Post-it notes to ensure that different persons would share their ideas and talk. Extensive notes were taken during the sessions, and all the Post-it notes were collected. A thematic content analysis was carried out to obtain a detailed written report and perform a content analysis.

## Results

### Development of the Makasi intervention

#### Makasi theory of intervention

The mobile team and peer community advisory group made important contributions concerning recruitment strategies and intervention content and structure. First, the mobile team identified optimal locations in the greater Paris area to implement the Makasi intervention (frequency of visitation, profile of people seen), proposed transporting tickets to participants to ensure follow-up, and provided input for communication tools. Second, the workshops with the community advisory group led to several conclusions regarding the contents of the intervention. According to the peer groups, the diversity of prevention tools was not well-known in the community, and the intervention needed to bring baseline knowledge and competency on topics regarding sexual health. They also insisted that the intervention could not “only” tackle sexual health; it had to address the material needs of migrants. Because the persons we sought to reach were experiencing profound social hardship, the intervention needed to provide some “*tangible*” or concrete material goods resources to participants that were directly and immediately useful to them. The peer groups also had three important ideas regarding the technique of the intervention. First, there was the importance of the community-based aspect for hard-to-reach populations. As a peer said, *“If it’s a white woman who comes with her files, it’s not going to work”*. Second, they insisted on the importance of delivering positive messages to empower participants. Eventually, they insisted on the fact that the van and stand of the CBO should not be associated with HIV; therefore, new materials and posters were necessary. Based on these insights, the Makasi intervention was thus defined as an intervention that starts with the persons’ capacities and strengths and helps them find appropriate existing resources and information while also obtaining knowledge on sexual health.

To better describe the theory of the intervention, we used Chen’s framework [31] to distinguish between the theory of the intervention (how the intervention works) and the theory of change (how the intervention is supposed to impact empowerment in sexual health). This framework provides a standardised way to present the requisites of a programme in the evaluation field, identifying the crucial elements of the intervention and the actors necessary for its implementation.

**Fig. 1:**
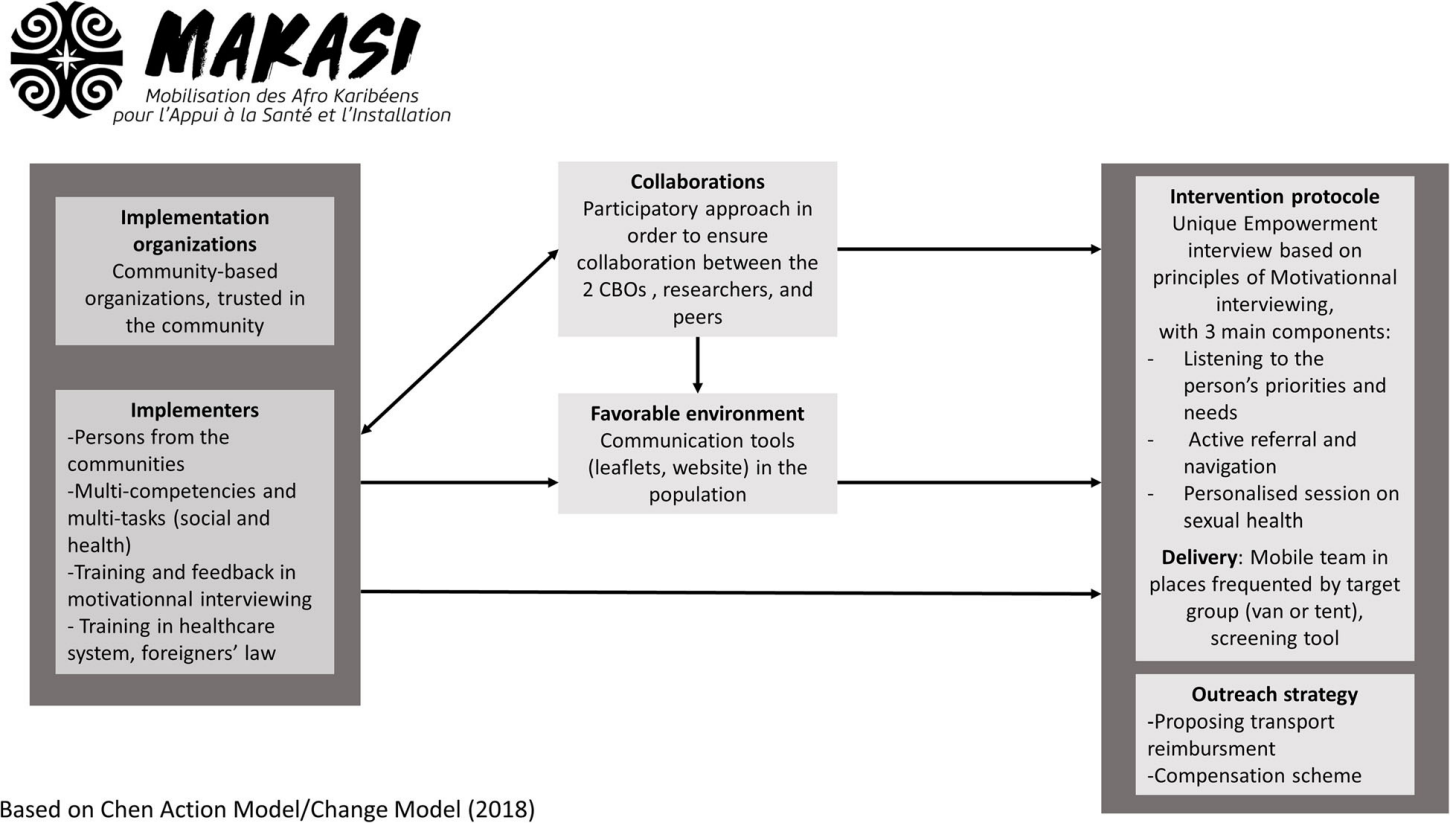
Makasi theory of intervention, 2019

Figure 1 presents the Makasi theory of intervention. A mobile team of health mediators routinely proposed rapid HIV testing and provided information about general health in public spaces where sub-Saharan and Caribbean immigrants are (markets and metro and suburban line stations). For all persons who visited the mobile team, there was a systematic rapid screening of the persons’ social and prevention needs, which took place in a confidential area, in a van or a little tent. The eligibility criteria were as follows: being 18 years old or older, being born in a country in sub-Saharan Africa or the non-French Caribbean and meeting at least one of the seven criteria of vulnerability: having unstable housing, being unemployed, having food deprivation, being undocumented, experiencing violence, having no medical insurance, and not knowing where to go to see a doctor. Eligible individuals were invited to participate in the Makasi intervention, specifically to meet with a social worker and engage in the empowerment-based interview; all participants provided written informed consent prior to participation.

The empowerment interview consisted of a unique 30-min intervention. It included listening to the person and helping her prioritise her social and health needs with the techniques of motivational interviewing. Second, the active referral and navigation system consisted of identifying the best place to refer the person according to her expressed needs. During the pilot, a mapping of the existing resources in the territory was performed, and contacts were made so that the person sent by the Makasi intervention would not find a closed door. Active referral also included calling the structure if necessary, giving the person an introduction letter to facilitate access and explaining to her how the structure works and what could be expected to happen, to reinforce participants’ autonomy. Finally, a personalised session on sexual health was delivered.

#### Makasi theory of change

**Fig. 2:**
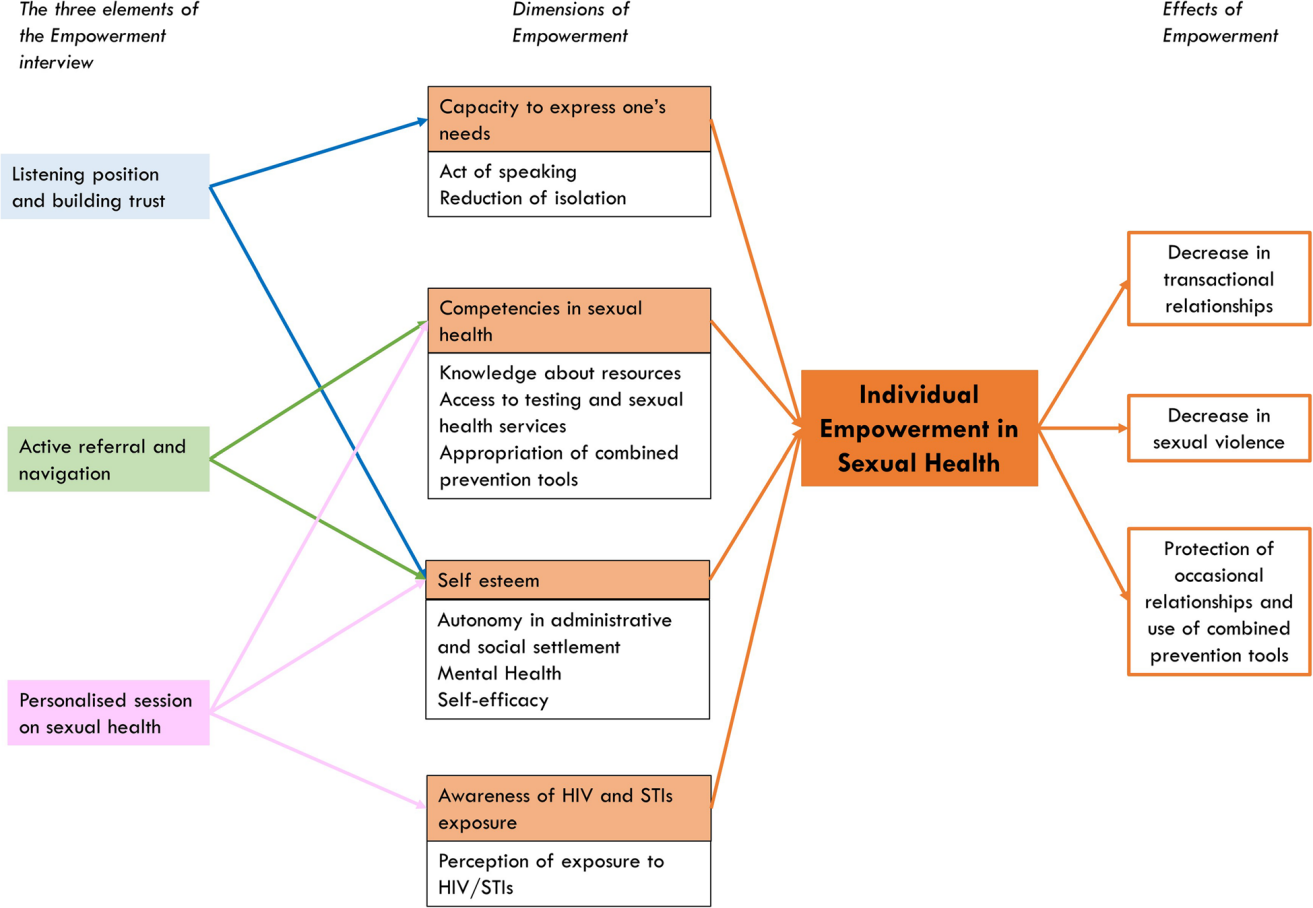
Makasi theory of change, 2019

The theory of change refers to the way the intervention is supposed to impact on desirable outcomes. The empowerment theory used in the Makasi intervention was based upon the four dimensions of Ninacs’ model of individual empowerment: 1) capacity to express one’s needs, 2) competencies and skills, 3) self-esteem and 4) critical conscience [32, 33]. This empowerment theory provided a complete framework in which the dimensions of individual empowerment used in other studies could be integrated [22, 23, 34]. Due to the participatory process, Ninacs’ framework was adapted for a sexual health context to obtain the Makasi theory of change (Fig. 2).

### Feasibility of the intervention

**Fig. 3:**
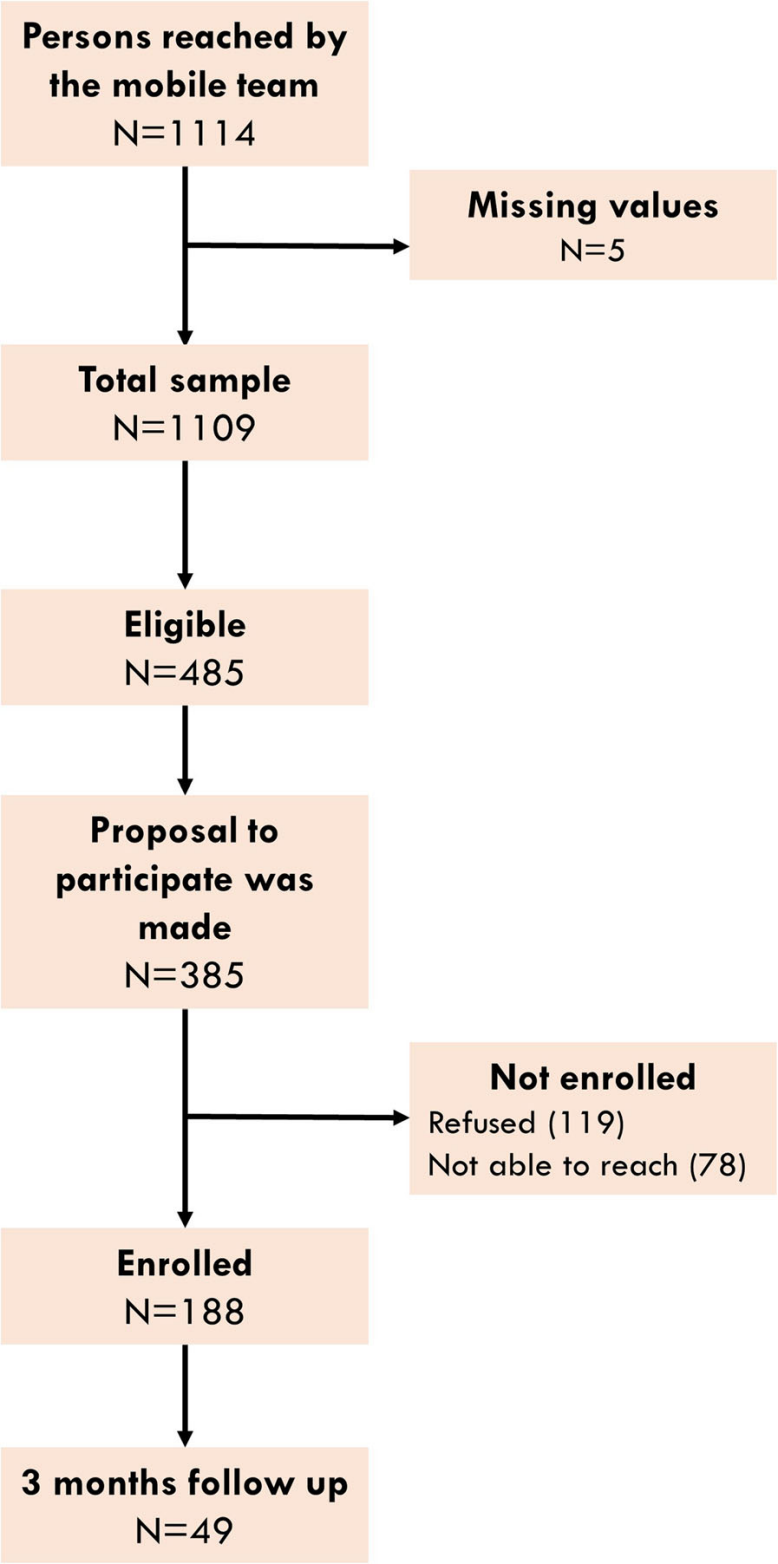
Flow chart of enrolment, Makasi pilot study 2018

Between April and December 2018, a total of 485 persons were identified as eligible using the screening questionnaire (Fig. 3). Participation in the intervention was proposed to 79% of eligible persons. When proposed, the persons agreed to participate in the intervention with a response rate of 69%. Some were lost to follow up, and 188 persons were finally included.

Table 1 provides information on the characteristics of the eligible persons who were asked to participate and of the participants in the Makasi intervention.

**Table 1:** Characteristics of eligible persons, according to participation status, Makasi pilot phase April–December 2018

	All (*n* = 1114)	Eligible (*n* = 485)	Proposal (*n* = 385)	Enrolled (*n* = 188)	Not enrolled (*n* = 197)	*p value*
Age						
Years (Mean)	35	35	34	34	35	
Gender						
Female %	33%	32%	30%	22%	39%	0.000
Region of birth						
West Africa	37%	60%	62%	63%	62%	0.277
Central Africa	23%	32%	32%	32%	32%	
Eastern and Southern Africa	3%	4%	3%	4%	2%	
Caribbean’s	4%	5%	3%	2%	4%	
Administrative situation						
% undocumented	31%	61%	70%	80%	60%	0.000
Employment situation						
% unemployed	39%	68%	71%	72%	69%	0.477

*p*-value: chi2 tests of comparison between enrolled and not enrolled

At enrolment, women are more often lost to follow up than men. Undocumented immigrants were more often enrolled. There was no difference in enrolment rates between immigrants from sub-Saharan Africa and those from the Caribbean.

### Feasibility of the stepped-wedge evaluation

The proportions of eligible individuals (45 and 42%) and of individuals agreeing to participate (65 and 74%) were similar and not significantly different in the immediate and deferred arms, respectively.

Three months after inclusion, persons were contacted by phone and text messages to meet for the post-intervention questionnaire (immediate arm) or to have the intervention delivered (deferred arm).

The retention rate at 3 months was 26% (*n* = 49) in the pilot phase. The persons who stayed in the Makasi intervention were not different from those who were lost at the 3-month follow up, except regarding sex (12% of women remained enrolled vs 29% of men). There was no difference in retention between the two arms.

### Insights from the reflexive process: how to improve the intervention?

The first reflexive workshop was held on December 18, 2018, for 4 h. This workshop gathered 29 participants (10 members of the research team, 5 peers, and 14 NGO members), and participatory methods allowed the team to find solutions for the main challenges identified. A second workshop took place on January 16th for 3 h to propose decisions on which ideas and solutions were to be kept. A final three-hour workshop on 25th January allowed the team to share the adopted solutions with the mobile team and to make final decisions about changes to be made to the intervention, the recruitment and the follow-up strategy. To address the low response rate at 3 months, additional personnel were immediately hired. Additionally, more contact details were collected at the time written consent was obtained. Third, the compensation that was originally given at the end of the whole process was now given at each step of the process (baseline and 3, 6 and 9 months) for a total of 40 euros. To improve participation, new communication tools were developed, and new locations were targeted.

Finally, the participatory process also led to the redaction of a Makasi intervention guide with a timed outline of the empowerment interview and practical sections with wording, main referrals, and key prevention and sexual health messages, with a reinforced focus on the person’s existing resources to enable change. Finally, the participative process suggested that for some participants, the interview and the referral tools were not enough: navigation, and physical navigation in particular, is needed to actually go to the structures and start the settlement or prevention process.

## Discussion

The objective of this paper was to describe the development of a community-based and context-adapted intervention aimed at improving sub-Saharan and Caribbean immigrants’ empowerment in sexual health, and to report results of the feasibility of the intervention and its evaluation design. We demonstrated that this process successfully led to the design of an intervention that addresses structural difficulties during settlement and that is based upon a conceptual model of empowerment and uses a reflexive process throughout. The community- and evidence-based outreach design of the intervention led us to successfully enrol a hard-to-reach population, largely undocumented and unemployed, in a prevention intervention. The tested design allowed us to precisely describe the characteristics of the reached, eligible and participant populations and to implement a controlled trial between an intervention arm (immediate) and a control arm (deferred).

Despite this success, the pilot phase of the study also highlighted several challenges that need to be addressed in the trial phase.

The first challenge is the low retention rate after three months, which makes the evaluation of the impact of the intervention difficult. In the existing literature [35, 36], we did not find studies conducted among healthy, non-drug-user immigrants with which we could compare our response rates. Overall, previous research highlights the difficulty of enrolling and retaining stigmatised populations [37], and people in general who go through social hardship experiences and who are very mobile, in research. In our case, the street-recruitment design of the intervention implies that there was no fixed place where people could go or call, although they were given a contact number at enrolment. The main solutions to improve retention rates that were identified in the literature were better collection of contact details at first inclusion [38], peer interviewers, staff training, going where the person is more comfortable to have the follow-up interview, and cash incentives [35, 38–40]. This evidence is thus very much in line with the results of the reflexive workshops. In addition, previous research notes the importance of acknowledging extended timeframes, planning for higher resourcing costs and operating via community partnerships [41].

A second challenge is the recruitment process that occurred in the Makasi intervention and resulted in an overrepresentation of men in the pilot. In general, women are more willing to participate in health studies [40–42], including sub-Saharan immigrants [43]. This has been explained by gender role differentiation, where women are in charge of health aspects and are closer to the health system because of contraception, pregnancy and childcare. However, in the Makasi intervention, not only does the CBO routinely reach fewer women but women were also less likely to participate and be present at follow up. Possible explanations for this are i) that it is difficult for women to ask questions about sexual health in public spaces because they fear to be disapproved of, as reported by the Afrique Avenir CBO workers and coherent with other studies on street-based HIV interventions [44] ii), that there is a lower presence of women in public spaces in general [45] , and iii) that there is less time to spend on the stand because women are busier with domestic work than men. Interestingly, the peer groups anticipated this last challenge, noting that “men have the time” and “the woman has to hurry”.

Finally, this pilot phase allowed us to test the feasibility of a rigorous, mixed-method stepped-wedge evaluation design. This was crucial as many community-based interventions exist but are seldom documented and evaluated according to evidence-based criteria.

Although no difference in enrolment was noted between Caribbean and sub-Saharan African immigrants, it should be noted that one of the difficulties of the project was that there are scarce data on Caribbean immigrants’ situations in France. It makes it difficult to put our results into perspective, and particular attention should be paid during the scale-up phase to examine whether the intervention is also relevant to Caribbean population needs.

An important limitation of this study is that although these results were thoroughly discussed with NGO members and peers, we were not able to present and discuss them with actual participants of the Makasi intervention. However, qualitative research is currently being done to collect and analyse participants’ discourses about the intervention and its consequences.

## Conclusion

At a time when immigrants experiencing harsh difficulties are under scrutiny in Europe, we describe how, in the greater Paris area, a participatory approach was used to design and evaluate the feasibility of a community-based intervention to improve sub-Saharan immigrants’ empowerment in sexual health. The pilot phase was key to building the participatory approach, ensuring that the intervention and its design would be adapted to the population and testing whether a controlled two-arm design was feasible. The findings concerning the response rates at baseline and follow up were not completely satisfactory and led to several adaptations of the protocol, including a further enhancement of the participatory and community-based approach and additional retention strategies. The next step of the project includes the trial phase, which will allow us to measure the impact of the newly developed Makasi intervention on sub-Saharan and Caribbean immigrants’ empowerment in sexual health.

## Supplementary information

**Additional file 1.** Makasi evaluation design, 2019

## Data Availability

All data available on request to corresponding author, Anne Gosselin anne.gosselin@icmigrations.fr

## Abbreviations

CBO: Community-based organisation
HIV: Human Immunodeficiency Virus
NGO: Non-Governmental Organisation
PrEP: Pre-Exposure Prophylaxis
STI: Sexually Transmitted Infection
