# Supplementary figures and images for: Participatory development and pilot testing of the Makasi intervention: a community-based outreach intervention to improve sub-Saharan and Caribbean immigrants’ empowerment in sexual health
Source: BMC Public Health. 2019 Dec 5;19:1646. doi: 10.1186/s12889-019-7943-2 (PMC6896752; doi:10.1186/s12889-019-7943-2)

**Supplementary Material 1. Makasi evaluation design, 2019**


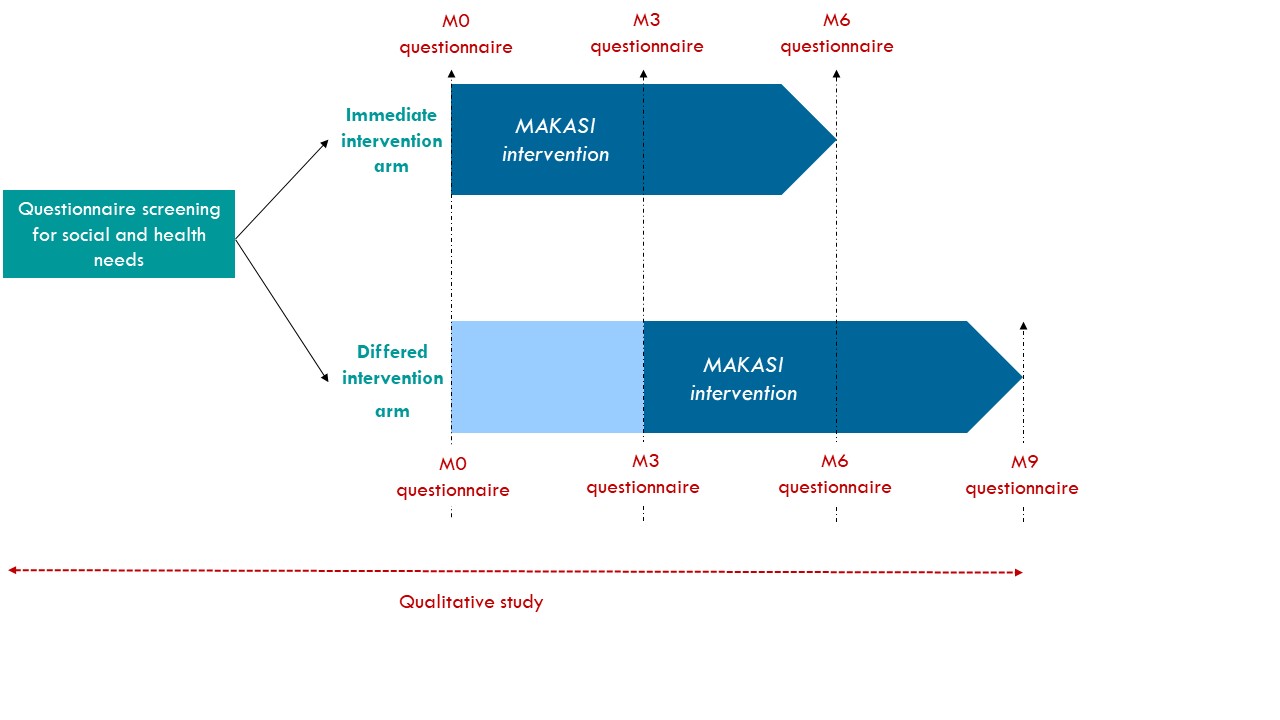

Supplement: Supplementary file 1 — Additional file 1. Makasi evaluation design, 2019 [file 12889_2019_7943_MOESM1_ESM.docx]
